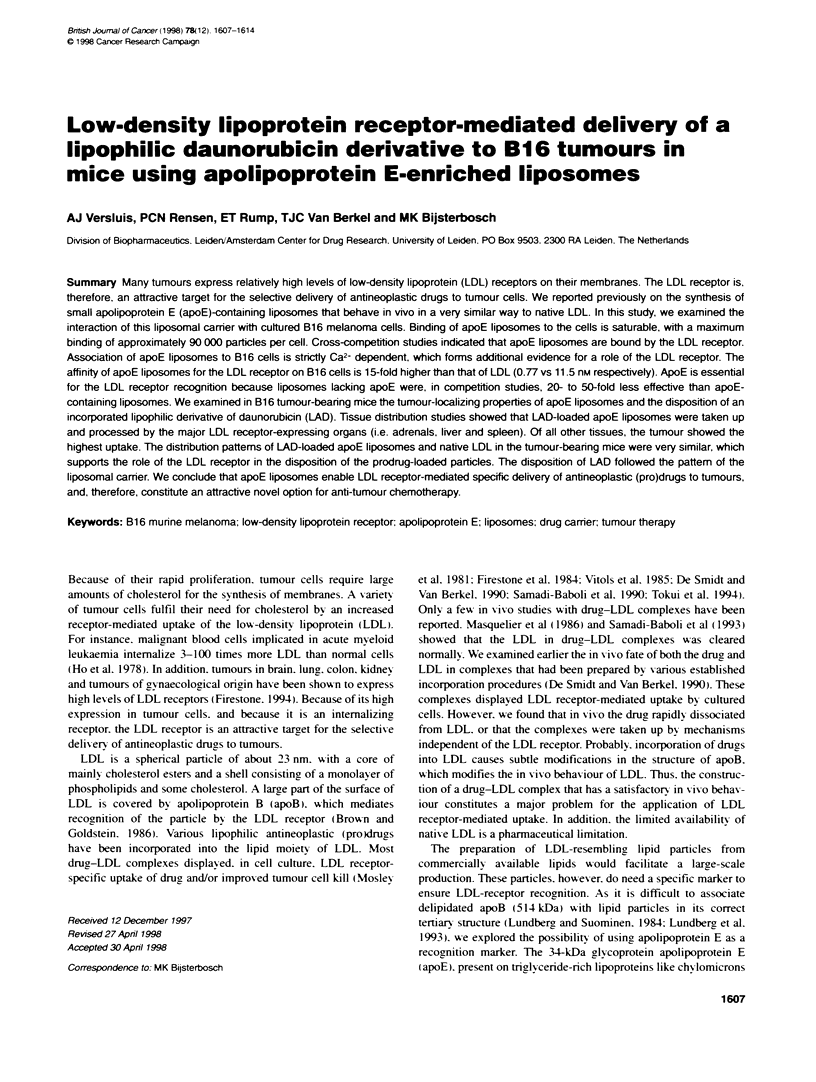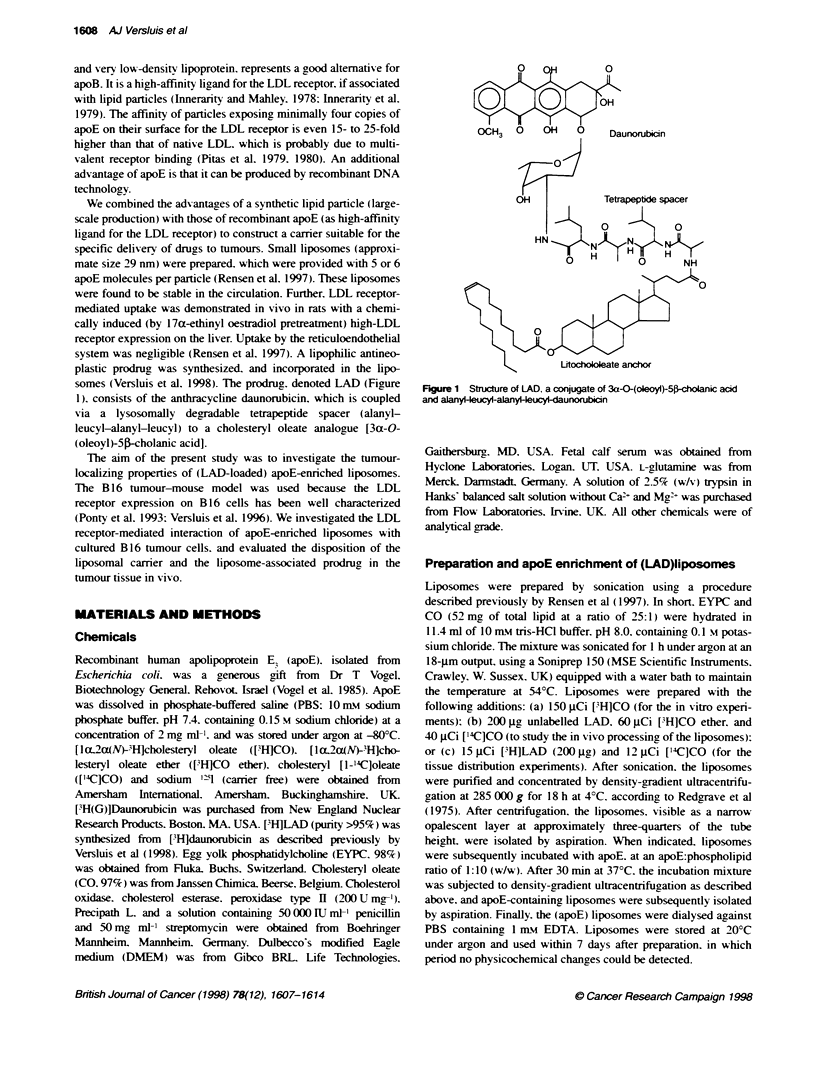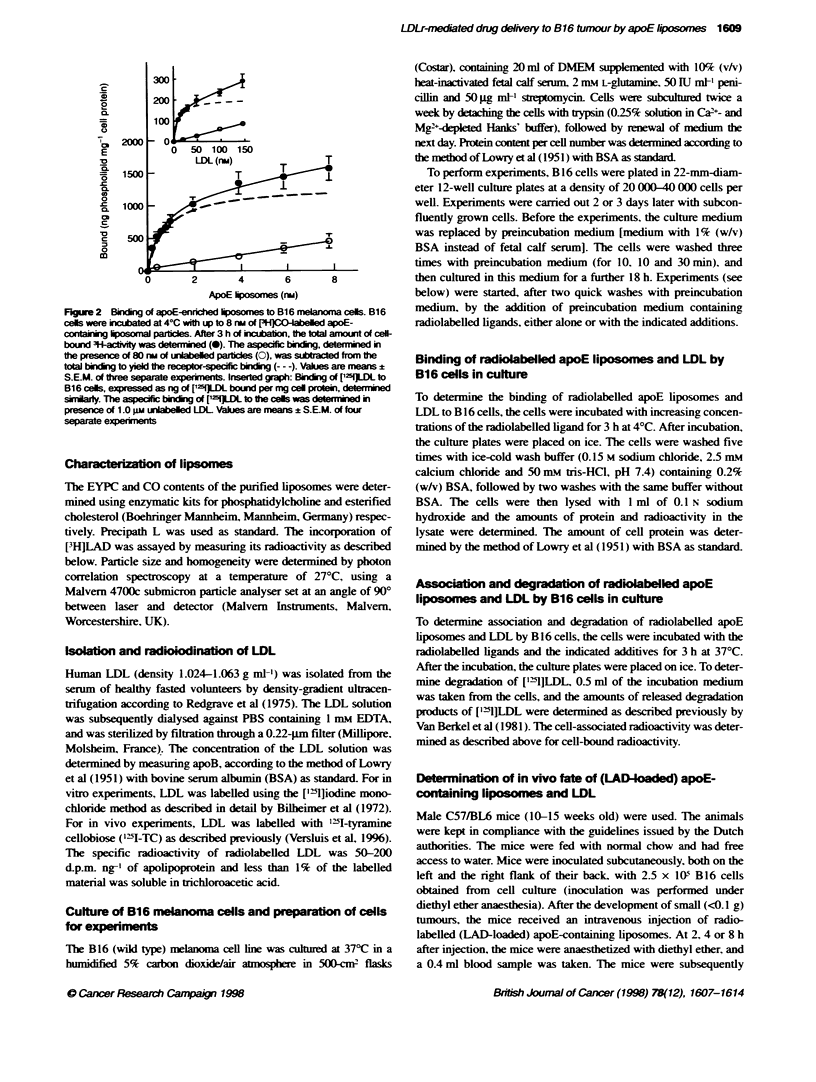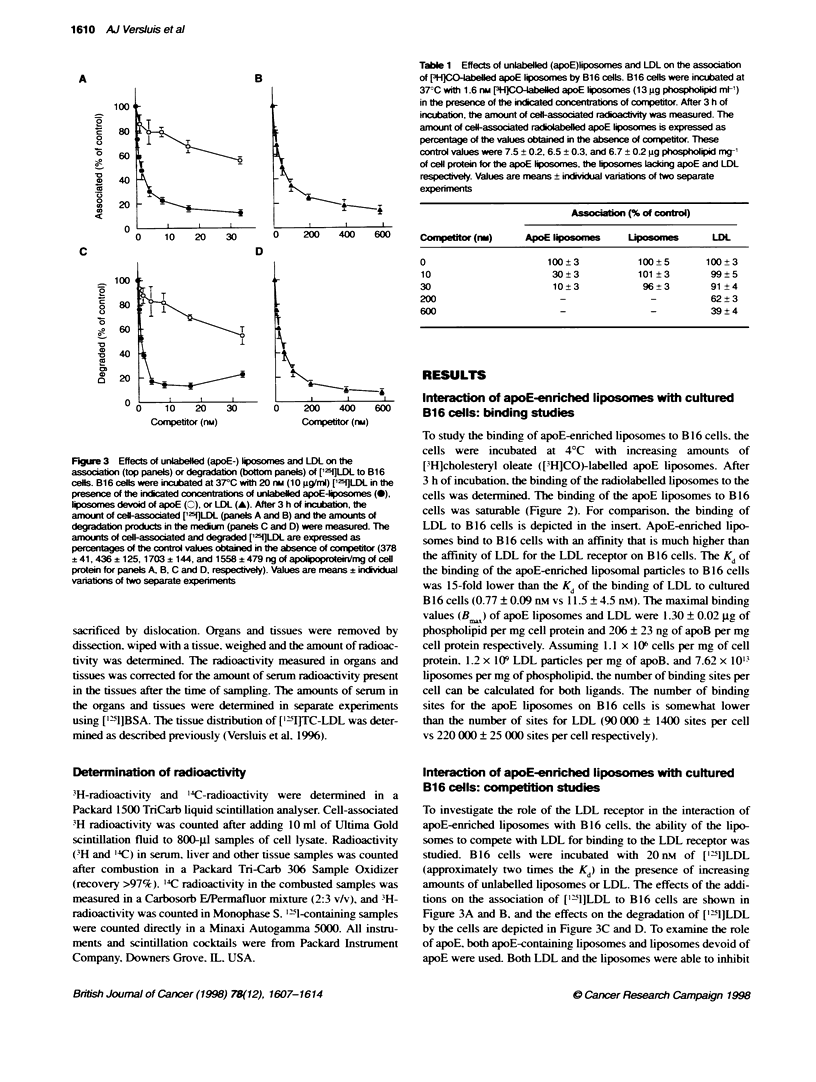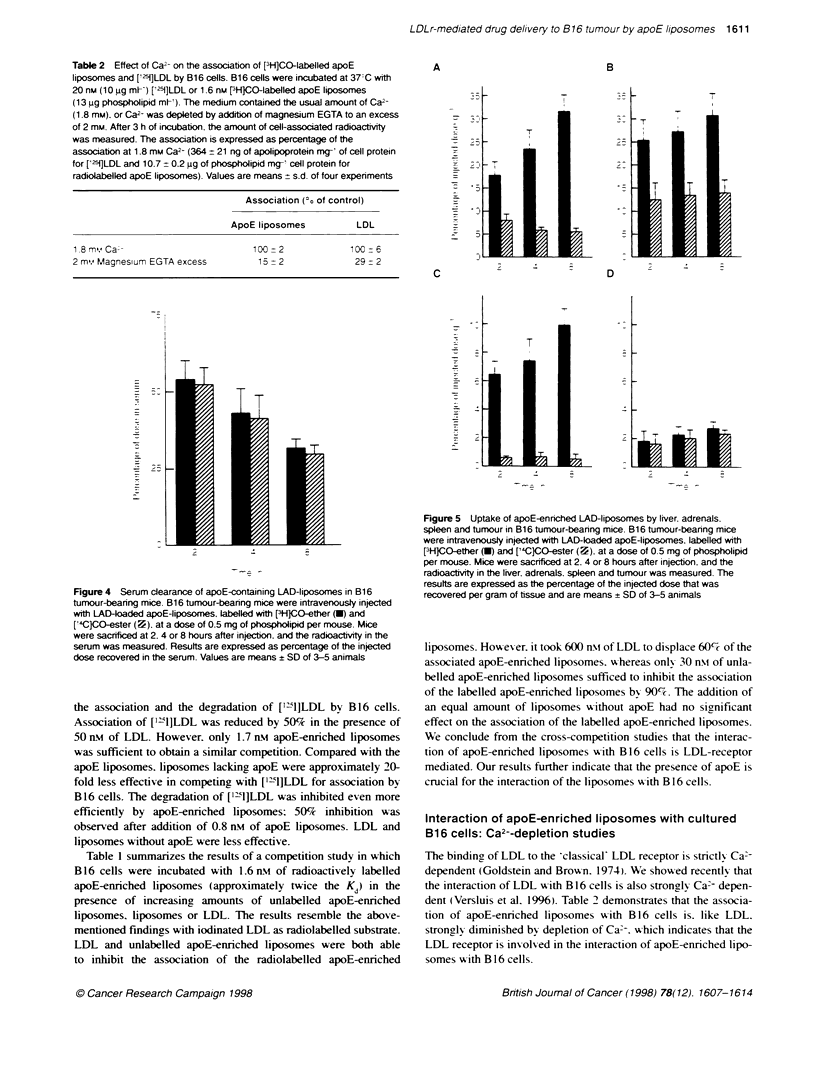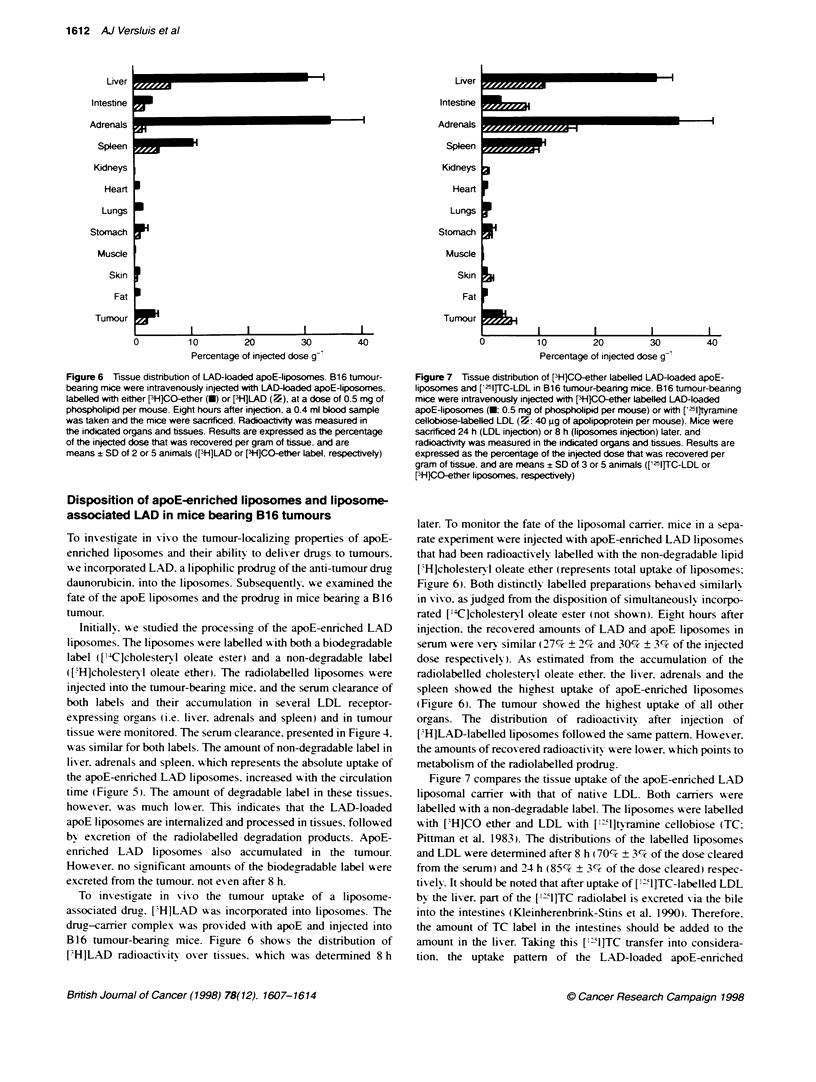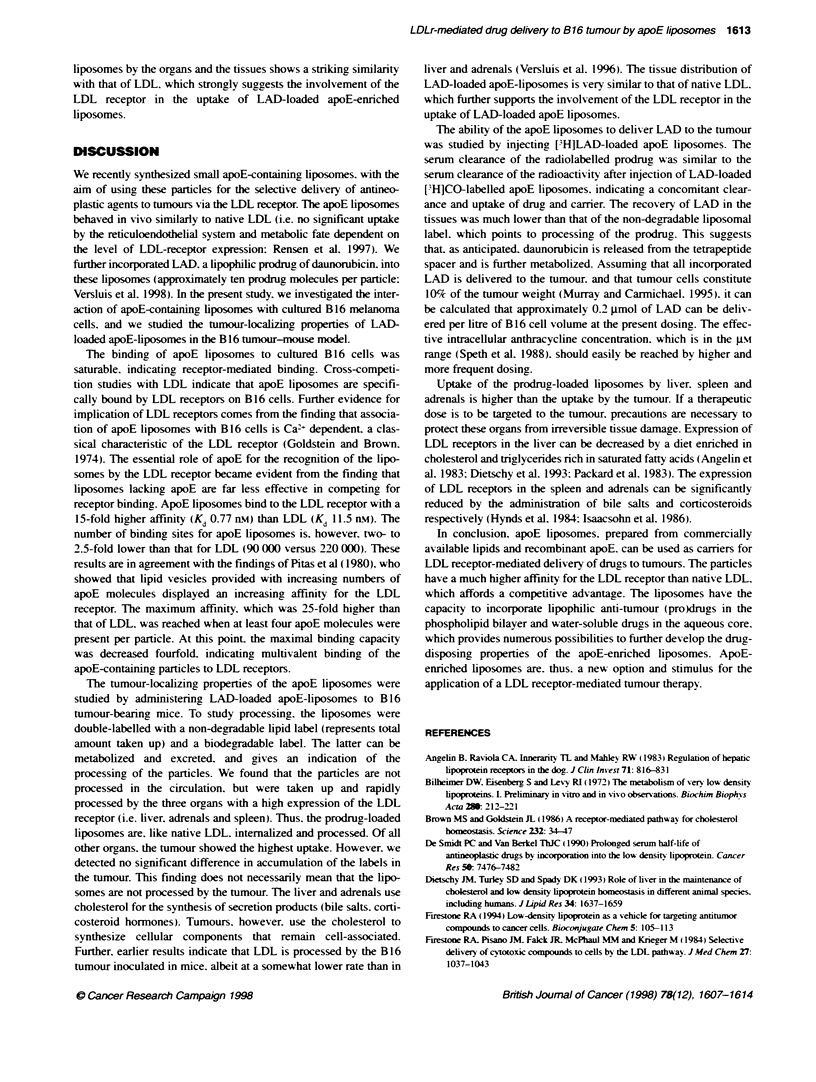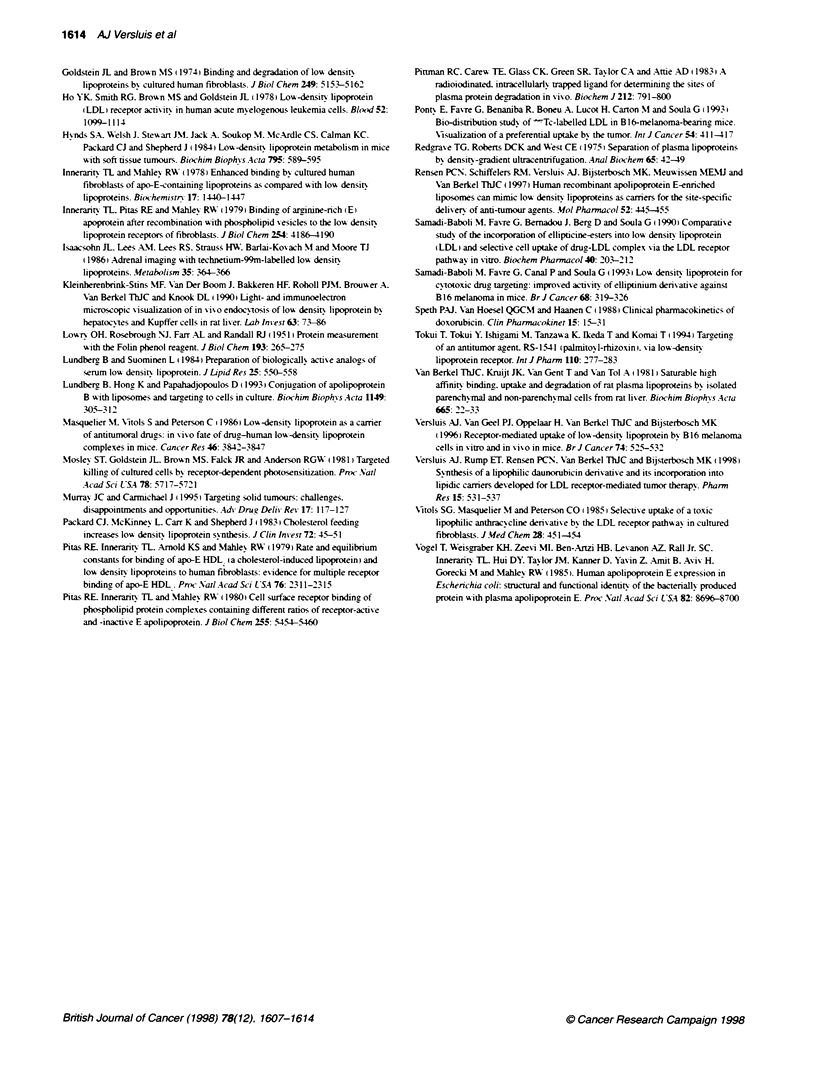# Low-density lipoprotein receptor-mediated delivery of a lipophilic daunorubicin derivative to B16 tumours in mice using apolipoprotein E-enriched liposomes.

**DOI:** 10.1038/bjc.1998.730

**Published:** 1998-12

**Authors:** A. J. Versluis, P. C. Rensen, E. T. Rump, T. J. Van Berkel, M. K. Bijsterbosch

**Affiliations:** Division of Biopharmaceutics, Leiden/Amsterdam Center for Drug Research, University of Leiden, The Netherlands.

## Abstract

Many tumours express relatively high levels of low-density lipoprotein (LDL) receptors on their membranes. The LDL receptor is, therefore, an attractive target for the selective delivery of antineoplastic drugs to tumour cells. We reported previously on the synthesis of small apolipoprotein E (apoE)-containing liposomes that behave in vivo in a very similar way to native LDL. In this study, we examined the interaction of this liposomal carrier with cultured B16 melanoma cells. Binding of apoE liposomes to the cells is saturable, with a maximum binding of approximately 90000 particles per cell. Cross-competition studies indicated that apoE liposomes are bound by the LDL receptor. Association of apoE liposomes to B16 cells is strictly Ca2+ dependent, which forms additional evidence for a role of the LDL receptor. The affinity of apoE liposomes for the LDL receptor on B16 cells is 15-fold higher than that of LDL (0.77 vs 11.5 nM respectively). ApoE is essential for the LDL receptor recognition because liposomes lacking apoE were, in competition studies, 20- to 50-fold less effective than apoE-containing liposomes. We examined in B16 tumour-bearing mice the tumour-localizing properties of apoE liposomes and the disposition of an incorporated lipophilic derivative of daunorubicin (LAD). Tissue distribution studies showed that LAD-loaded apoE liposomes were taken up and processed by the major LDL receptor-expressing organs (i.e. adrenals, liver and spleen). Of all other tissues, the tumour showed the highest uptake. The distribution patterns of LAD-loaded apoE liposomes and native LDL in the tumour-bearing mice were very similar, which supports the role of the LDL receptor in the disposition of the prodrug-loaded particles. The disposition of LAD followed the pattern of the liposomal carrier. We conclude that apoE liposomes enable LDL receptor-mediated specific delivery of antineoplastic (pro)drugs to tumours, and, therefore, constitute an attractive novel option for anti-tumour chemotherapy.


British Journal of Cancer (1998) 78(12), 1607–1614
© 1998 Cancer Research Campaign

### Low-density lipoprotein receptor-mediated delivery of a lipophilic daunorubicin derivative to B16 tumours in mice using apolipoprotein E-enriched liposomes

**AJ Versluis, PCN Rensen, ET Rump, TJC Van Berkel and MK Bijsterbosch**

Division of Biopharmaceutics, Leiden/Amsterdam Center for Drug Research, University of Leiden, PO Box 9503, 2300 RA Leiden, The Netherlands

**Summary** Many tumours express relatively high levels of low-density lipoprotein (LDL) receptors on their membranes. The LDL receptor is, therefore, an attractive target for the selective delivery of antineoplastic drugs to tumour cells. We reported previously on the synthesis of small apolipoprotein E (apoE)-containing liposomes that behave in vivo in a very similar way to native LDL. In this study, we examined the interaction of this liposomal carrier with cultured B16 melanoma cells. Binding of apoE liposomes to the cells is saturable, with a maximum binding of approximately 90 000 particles per cell. Cross-competition studies indicated that apoE liposomes are bound by the LDL receptor. Association of apoE liposomes to B16 cells is strictly $Ca^{2+}$ dependent, which forms additional evidence for a role of the LDL receptor. The affinity of apoE liposomes for the LDL receptor on B16 cells is 15-fold higher than that of LDL (0.77 vs 11.5 nM respectively). ApoE is essential for the LDL receptor recognition because liposomes lacking apoE were, in competition studies, 20- to 50-fold less effective than apoE-containing liposomes. We examined in B16 tumour-bearing mice the tumour-localizing properties of apoE liposomes and the disposition of an incorporated lipophilic derivative of daunorubicin (LAD). Tissue distribution studies showed that LAD-loaded apoE liposomes were taken up and processed by the major LDL receptor-expressing organs (i.e. adrenals, liver and spleen). Of all other tissues, the tumour showed the highest uptake. The distribution patterns of LAD-loaded apoE liposomes and native LDL in the tumour-bearing mice were very similar, which supports the role of the LDL receptor in the disposition of the prodrug-loaded particles. The disposition of LAD followed the pattern of the liposomal carrier. We conclude that apoE liposomes enable LDL receptor-mediated specific delivery of antineoplastic (pro)drugs to tumours, and, therefore, constitute an attractive novel option for anti-tumour chemotherapy.

**Keywords:** B16 murine melanoma; low-density lipoprotein receptor; apolipoprotein E; liposomes; drug carrier; tumour therapy

Because of their rapid proliferation, tumour cells require large amounts of cholesterol for the synthesis of membranes. A variety of tumour cells fulfil their need for cholesterol by an increased receptor-mediated uptake of the low-density lipoprotein (LDL). For instance, malignant blood cells implicated in acute myeloid leukaemia internalize 3–100 times more LDL than normal cells (Ho et al, 1978). In addition, tumours in brain, lung, colon, kidney and tumours of gynaecological origin have been shown to express high levels of LDL receptors (Firestone, 1994). Because of its high expression in tumour cells, and because it is an internalizing receptor, the LDL receptor is an attractive target for the selective delivery of antineoplastic drugs to tumours.

LDL is a spherical particle of about 23 nm, with a core of mainly cholesterol esters and a shell consisting of a monolayer of phospholipids and some cholesterol. A large part of the surface of LDL is covered by apolipoprotein B (apoB), which mediates recognition of the particle by the LDL receptor (Brown and Goldstein, 1986). Various lipophilic antineoplastic (pro)drugs have been incorporated into the lipid moiety of LDL. Most drug–LDL complexes displayed, in cell culture, LDL receptor-specific uptake of drug and/or improved tumour cell kill (Mosley et al, 1981; Firestone et al, 1984; Vitols et al, 1985; De Smidt and Van Berkel, 1990; Samadi-Baboli et al, 1990; Tokui et al, 1994). Only a few in vivo studies with drug–LDL complexes have been reported. Masquelier et al (1986) and Samadi-Baboli et al (1993) showed that the LDL in drug–LDL complexes was cleared normally. We examined earlier the in vivo fate of both the drug and LDL in complexes that had been prepared by various established incorporation procedures (De Smidt and Van Berkel, 1990). These complexes displayed LDL receptor-mediated uptake by cultured cells. However, we found that in vivo the drug rapidly dissociated from LDL, or that the complexes were taken up by mechanisms independent of the LDL receptor. Probably, incorporation of drugs into LDL causes subtle modifications in the structure of apoB, which modifies the in vivo behaviour of LDL. Thus, the construction of a drug–LDL complex that has a satisfactory in vivo behaviour constitutes a major problem for the application of LDL receptor-mediated uptake. In addition, the limited availability of native LDL is a pharmaceutical limitation.

The preparation of LDL-resembling lipid particles from commercially available lipids would facilitate a large-scale production. These particles, however, do need a specific marker to ensure LDL-receptor recognition. As it is difficult to associate delipidated apoB (514 kDa) with lipid particles in its correct tertiary structure (Lundberg and Suominen, 1984; Lundberg et al, 1993), we explored the possibility of using apolipoprotein E as a recognition marker. The 34-kDa glycoprotein apolipoprotein E (apoE), present on triglyceride-rich lipoproteins like chylomicrons

*Received 12 December 1997*
*Revised 27 April 1998*
*Accepted 30 April 1998*

*Correspondence to:* MK Bijsterbosch and very low-density lipoprotein, represents a good alternative for apoB. It is a high-affinity ligand for the LDL receptor, if associated with lipid particles (Innerarity and Mahley, 1978; Innerarity et al, 1979). The affinity of particles exposing minimally four copies of apoE on their surface for the LDL receptor is even 15- to 25-fold higher than that of native LDL, which is probably due to multivalent receptor binding (Pitas et al, 1979, 1980). An additional advantage of apoE is that it can be produced by recombinant DNA technology.

We combined the advantages of a synthetic lipid particle (large-scale production) with those of recombinant apoE (as high-affinity ligand for the LDL receptor) to construct a carrier suitable for the specific delivery of drugs to tumours. Small liposomes (approximate size 29 nm) were prepared, which were provided with 5 or 6 apoE molecules per particle (Rensen et al, 1997). These liposomes were found to be stable in the circulation. Further, LDL receptor-mediated uptake was demonstrated in vivo in rats with a chemically induced (by 17α-ethinyl oestradiol pretreatment) high-LDL receptor expression on the liver. Uptake by the reticuloendothelial system was negligible (Rensen et al, 1997). A lipophilic antineoplastic prodrug was synthesized, and incorporated in the liposomes (Versluis et al, 1998). The prodrug, denoted LAD (Figure 1), consists of the anthracycline daunorubicin, which is coupled via a lysosomally degradable tetrapeptide spacer (alanyl–leucyl–alanyl–leucyl) to a cholesteryl oleate analogue [3α-*O*-(oleoyl)-5β-cholanic acid].

The aim of the present study was to investigate the tumour-localizing properties of (LAD-loaded) apoE-enriched liposomes. The B16 tumour–mouse model was used because the LDL receptor expression on B16 cells has been well characterized (Ponty et al, 1993; Versluis et al, 1996). We investigated the LDL receptor-mediated interaction of apoE-enriched liposomes with cultured B16 tumour cells, and evaluated the disposition of the liposomal carrier and the liposome-associated prodrug in the tumour tissue in vivo.

**Figure 1** Structure of LAD, a conjugate of 3α-O-(oleoyl)-5β-cholanic acid and alanyl-leucyl-alanyl-leucyl-daunorubicin

### MATERIALS AND METHODS

### Chemicals

Recombinant human apolipoprotein $E_3$ (apoE), isolated from *Escherichia coli*, was a generous gift from Dr T Vogel, Biotechnology General, Rehovot, Israel (Vogel et al, 1985). ApoE was dissolved in phosphate-buffered saline (PBS: 10 mM sodium phosphate buffer, pH 7.4, containing 0.15 M sodium chloride) at a concentration of 2 mg $ml^{-1}$, and was stored under argon at -80°C. $[1\alpha,2\alpha(N)\text{-}^{3}H]$cholesteryl oleate ($[^{3}H]$CO), $[1\alpha,2\alpha(N)\text{-}^{3}H]$cholesteryl oleate ether ($[^{3}H]$CO ether), cholesteryl $[1\text{-}^{14}C]$oleate ($[^{14}C]$CO) and sodium $^{125}I$ (carrier free) were obtained from Amersham International, Amersham, Buckinghamshire, UK. $[^{3}H(G)]$Daunorubicin was purchased from New England Nuclear Research Products, Boston, MA, USA. $[^{3}H]$LAD (purity >95%) was synthesized from $[^{3}H]$daunorubicin as described previously by Versluis et al (1998). Egg yolk phosphatidylcholine (EYPC, 98%) was obtained from Fluka, Buchs, Switzerland. Cholesteryl oleate (CO, 97%) was from Janssen Chimica, Beerse, Belgium. Cholesterol oxidase, cholesterol esterase, peroxidase type II (200 U $mg^{-1}$), Precipath L, and a solution containing 50 000 IU $ml^{-1}$ penicillin and 50 mg $ml^{-1}$ streptomycin were obtained from Boehringer Mannheim, Mannheim, Germany. Dulbecco's modified Eagle medium (DMEM) was from Gibco BRL, Life Technologies, Gaithersburg, MD, USA. Fetal calf serum was obtained from Hyclone Laboratories, Logan, UT, USA. L-glutamine was from Merck, Darmstadt, Germany. A solution of 2.5% (w/v) trypsin in Hanks' balanced salt solution without $Ca^{2+}$ and $Mg^{2+}$ was purchased from Flow Laboratories, Irvine, UK. All other chemicals were of analytical grade.

### Preparation and apoE enrichment of (LAD)liposomes

Liposomes were prepared by sonication using a procedure described previously by Rensen et al (1997). In short, EYPC and CO (52 mg of total lipid at a ratio of 25:1) were hydrated in 11.4 ml of 10 mM tris-HCl buffer, pH 8.0, containing 0.1 M potassium chloride. The mixture was sonicated for 1 h under argon at an 18-μm output, using a Soniprep 150 (MSE Scientific Instruments, Crawley, W. Sussex, UK) equipped with a water bath to maintain the temperature at 54°C. Liposomes were prepared with the following additions: (a) 150 μCi $[^{3}H]$CO (for the in vitro experiments); (b) 200 μg unlabelled LAD, 60 μCi $[^{3}H]$CO ether, and 40 μCi $[^{14}C]$CO (to study the in vivo processing of the liposomes); or (c) 15 μCi $[^{3}H]$LAD (200 μg) and 12 μCi $[^{14}C]$CO (for the tissue distribution experiments). After sonication, the liposomes were purified and concentrated by density-gradient ultracentrifugation at 285 000 *g* for 18 h at 4°C, according to Redgrave et al (1975). After centrifugation, the liposomes, visible as a narrow opalescent layer at approximately three-quarters of the tube height, were isolated by aspiration. When indicated, liposomes were subsequently incubated with apoE, at an apoE:phospholipid ratio of 1:10 (w/w). After 30 min at 37°C, the incubation mixture was subjected to density-gradient ultracentrifugation as described above, and apoE-containing liposomes were subsequently isolated by aspiration. Finally, the (apoE) liposomes were dialysed against PBS containing 1 mM EDTA. Liposomes were stored at 20°C under argon and used within 7 days after preparation, in which period no physicochemical changes could be detected.

 © Cancer Research Campaign 1998

**Figure 2** Binding of apoE-enriched liposomes to B16 melanoma cells. B16 cells were incubated at 4°C with up to 8 nM of [$^{3}$H]CO-labelled apoE-containing liposomal particles. After 3 h of incubation, the total amount of cell-bound $^{3}$H-activity was determined (●). The aspecific binding, determined in the presence of 80 nM of unlabelled particles (○), was subtracted from the total binding to yield the receptor-specific binding (- - -). Values are means ± S.E.M. of three separate experiments. Inserted graph: Binding of [$^{125}$I]LDL to B16 cells, expressed as ng of [$^{125}$I]LDL bound per mg cell protein, determined similarly. The aspecific binding of [$^{125}$I]LDL to the cells was determined in presence of 1.0 µM unlabelled LDL. Values are means ± S.E.M. of four separate experiments

### Characterization of lipsomes

The EYPC and CO contents of the purified liposomes were determined using enzymatic kits for phosphatidylcholine and esterified cholesterol (Boehringer Mannheim, Mannheim, Germany) respectively. Precipath L was used as standard. The incorporation of [$^{3}$H]LAD was assayed by measuring its radioactivity as described below. Particle size and homogeneity were determined by photon correlation spectroscopy at a temperature of 27°C, using a Malvern 4700c submicron particle analyser set at an angle of 90° between laser and detector (Malvern Instruments, Malvern, Worcestershire, UK).

### Isolation and radioiodination of LDL

Human LDL (density 1.024–1.063 g ml$^{-1}$) was isolated from the serum of healthy fasted volunteers by density-gradient ultracentrifugation according to Redgrave et al (1975). The LDL solution was subsequently dialysed against PBS containing 1 mM EDTA, and was sterilized by filtration through a 0.22-µm filter (Millipore, Molsheim, France). The concentration of the LDL solution was determined by measuring apoB, according to the method of Lowry et al (1951) with bovine serum albumin (BSA) as standard. For in vitro experiments, LDL was labelled using the [$^{125}$I]iodine monochloride method as described in detail by Bilheimer et al (1972). For in vivo experiments, LDL was labelled with $^{125}$I-tyramine cellobiose ($^{125}$I-TC) as described previously (Versluis et al, 1996). The specific radioactivity of radiolabelled LDL was 50–200 d.p.m. ng$^{-1}$ of apolipoprotein and less than 1% of the labelled material was soluble in trichloroacetic acid.

### Culture of B16 melanoma cells and preparation of cells for experiments

The B16 (wild type) melanoma cell line was cultured at 37°C in a humidified 5% carbon dioxide/air atmosphere in 500-cm$^{2}$ flasks (Costar), containing 20 ml of DMEM supplemented with 10% (v/v) heat-inactivated fetal calf serum, 2 mM L-glutamine, 50 IU ml$^{-1}$ penicillin and 50 µg ml$^{-1}$ streptomycin. Cells were subcultured twice a week by detaching the cells with trypsin (0.25% solution in $Ca^{2+}$- and $Mg^{2+}$-depleted Hanks' buffer), followed by renewal of medium the next day. Protein content per cell number was determined according to the method of Lowry et al (1951) with BSA as standard.

To perform experiments, B16 cells were plated in 22-mm-diameter 12-well culture plates at a density of 20 000–40 000 cells per well. Experiments were carried out 2 or 3 days later with subconfluently grown cells. Before the experiments, the culture medium was replaced by preincubation medium [medium with 1% (w/v) BSA instead of fetal calf serum]. The cells were washed three times with preincubation medium (for 10, 10 and 30 min), and then cultured in this medium for a further 18 h. Experiments (see below) were started, after two quick washes with preincubation medium, by the addition of preincubation medium containing radiolabelled ligands, either alone or with the indicated additions.

### Binding of radiolabelled apoE liposomes and LDL by B16 cells in culture

To determine the binding of radiolabelled apoE liposomes and LDL to B16 cells, the cells were incubated with increasing concentrations of the radiolabelled ligand for 3 h at 4°C. After incubation, the culture plates were placed on ice. The cells were washed five times with ice-cold wash buffer (0.15 M sodium chloride, 2.5 mM calcium chloride and 50 mM tris-HCl, pH 7.4) containing 0.2% (w/v) BSA, followed by two washes with the same buffer without BSA. The cells were then lysed with 1 ml of 0.1 N sodium hydroxide and the amounts of protein and radioactivity in the lysate were determined. The amount of cell protein was determined by the method of Lowry et al (1951) with BSA as standard.

### Association and degradation of radiolabelled apoE liposomes and LDL by B16 cells in culture

To determine association and degradation of radiolabelled apoE liposomes and LDL by B16 cells, the cells were incubated with the radiolabelled ligands and the indicated additives for 3 h at 37°C. After the incubation, the culture plates were placed on ice. To determine degradation of [$^{125}$I]LDL, 0.5 ml of the incubation medium was taken from the cells, and the amounts of released degradation products of [$^{125}$I]LDL were determined as described previously by Van Berkel et al (1981). The cell-associated radioactivity was determined as described above for cell-bound radioactivity.

### Determination of in vivo fate of (LAD-loaded) apoE-containing liposomes and LDL

Male C57/BL6 mice (10–15 weeks old) were used. The animals were kept in compliance with the guidelines issued by the Dutch authorities. The mice were fed with normal chow and had free access to water. Mice were inoculated subcutaneously, both on the left and the right flank of their back, with 2.5 × 10$^{5}$ B16 cells obtained from cell culture (inoculation was performed under diethyl ether anaesthesia). After the development of small (<0.1 g) tumours, the mice received an intravenous injection of radiolabelled (LAD-loaded) apoE-containing liposomes. At 2, 4 or 8 h after injection, the mice were anaesthetized with diethyl ether, and a 0.4 ml blood sample was taken. The mice were subsequently

**Figure 3** Effects of unlabelled (apoE-) liposomes and LDL on the association (top panels) or degradation (bottom panels) of [$^{125}$I]LDL to B16 cells. B16 cells were incubated at 37°C with 20 nM (10 µg/ml) [$^{125}$I]LDL in the presence of the indicated concentrations of unlabelled apoE-liposomes (●), liposomes devoid of apoE (○), or LDL (▲). After 3 h of incubation, the amount of cell-associated [$^{125}$I]LDL (panels A and B) and the amounts of degradation products in the medium (panels C and D) were measured. The amounts of cell-associated and degraded [$^{125}$I]LDL are expressed as percentages of the control values obtained in the absence of competitor (378 ± 41, 436 ± 125, 1703 ± 144, and 1558 ± 479 ng of apolipoprotein/mg of cell protein for panels A, B, C and D, respectively). Values are means ± individual variations of two separate experiments sacrificed by dislocation. Organs and tissues were removed by dissection, wiped with a tissue, weighed and the amount of radioactivity was determined. The radioactivity measured in organs and tissues was corrected for the amount of serum radioactivity present in the tissues after the time of sampling. The amounts of serum in the organs and tissues were determined in separate experiments using [$^{125}$I]BSA. The tissue distribution of [$^{125}$I]TC-LDL was determined as described previously (Versluis et al, 1996).

### Determination of radioactivity

$^{3}$H-radioactivity and $^{14}$C-radioactivity were determined in a Packard 1500 TriCarb liquid scintillation analyser. Cell-associated $^{3}$H radioactivity was counted after adding 10 ml of Ultima Gold scintillation fluid to 800-µl samples of cell lysate. Radioactivity ($^{3}$H and $^{14}$C) in serum, liver and other tissue samples was counted after combustion in a Packard Tri-Carb 306 Sample Oxidizer (recovery >97%). $^{14}$C radioactivity in the combusted samples was measured in a Carbosorb E/Permafluor mixture (2:3 v/v), and $^{3}$H-radioactivity was counted in Monophase S. $^{125}$I-containing samples were counted directly in a Minaxi Autogamma 5000. All instruments and scintillation cocktails were from Packard Instrument Company, Downers Grove, IL, USA.

**Table 1** Effects of unlabelled (apoE)liposomes and LDL on the association of [$^{3}$H]CO-labelled apoE liposomes by B16 cells. B16 cells were incubated at 37°C with 1.6 nM [$^{3}$H]CO-labelled apoE liposomes (13 µg phospholipid ml$^{-1}$) in the presence of the indicated concentrations of competitor. After 3 h of incubation, the amount of cell-associated radioactivity was measured. The amount of cell-associated radiolabelled apoE liposomes is expressed as percentage of the values obtained in the absence of competitor. These control values were 7.5 ± 0.2, 6.5 ± 0.3, and 6.7 ± 0.2 µg phospholipid mg$^{-1}$ of cell protein for the apoE liposomes, the liposomes lacking apoE and LDL respectively. Values are means ± individual variations of two separate experiments

| | Association (% of control) | | |
|---|---|---|---|
| **Competitor (nM)** | **ApoE liposomes** | **Liposomes** | **LDL** |
| 0 | 100 ± 3 | 100 ± 5 | 100 ± 3 |
| 10 | 30 ± 3 | 101 ± 3 | 99 ± 5 |
| 30 | 10 ± 3 | 96 ± 3 | 91 ± 4 |
| 200 | – | – | 62 ± 3 |
| 600 | – | – | 39 ± 4 |

### RESULTS

### Interaction of apoE-enriched liposomes with cultured B16 cells: binding studies

To study the binding of apoE-enriched liposomes to B16 cells, the cells were incubated at 4°C with increasing amounts of [$^{3}$H]cholesteryl oleate ([$^{3}$H]CO)-labelled apoE liposomes. After 3 h of incubation, the binding of the radiolabelled liposomes to the cells was determined. The binding of the apoE liposomes to B16 cells was saturable (Figure 2). For comparison, the binding of LDL to B16 cells is depicted in the insert. ApoE-enriched liposomes bind to B16 cells with an affinity that is much higher than the affinity of LDL for the LDL receptor on B16 cells. The $K_d$ of the binding of the apoE-enriched liposomal particles to B16 cells was 15-fold lower than the $K_d$ of the binding of LDL to cultured B16 cells (0.77 ± 0.09 nM vs 11.5 ± 4.5 nM). The maximal binding values ($B_{max}$) of apoE liposomes and LDL were 1.30 ± 0.02 µg of phospholipid per mg cell protein and 206 ± 23 ng of apoB per mg cell protein respectively. Assuming 1.1 × 10$^{6}$ cells per mg of cell protein, 1.2 × 10$^{9}$ LDL particles per mg of apoB, and 7.62 × 10$^{13}$ liposomes per mg of phospholipid, the number of binding sites per cell can be calculated for both ligands. The number of binding sites for the apoE liposomes on B16 cells is somewhat lower than the number of sites for LDL (90 000 ± 1400 sites per cell vs 220 000 ± 25 000 sites per cell respectively).

### Interaction of apoE-enriched liposomes with cultured B16 cells: competition studies

To investigate the role of the LDL receptor in the interaction of apoE-enriched liposomes with B16 cells, the ability of the liposomes to compete with LDL for binding to the LDL receptor was studied. B16 cells were incubated with 20 nM of [$^{125}$I]LDL (approximately two times the $K_d$) in the presence of increasing amounts of unlabelled liposomes or LDL. The effects of the additions on the association of [$^{125}$I]LDL to B16 cells are shown in Figure 3A and B, and the effects on the degradation of [$^{125}$I]LDL by the cells are depicted in Figure 3C and D. To examine the role of apoE, both apoE-containing liposomes and liposomes devoid of apoE were used. Both LDL and the liposomes were able to inhibit

© Cancer Research Campaign 1998

**Table 2** Effect of $Ca^{2+}$ on the association of [$^{3}$H]CO-labelled apoE liposomes and [$^{125}$I]LDL by B16 cells. B16 cells were incubated at 37°C with 20 nM (10 μg ml$^{-1}$) [$^{125}$I]LDL or 1.6 nM [$^{3}$H]CO-labelled apoE liposomes (13 μg phospholipid ml$^{-1}$). The medium contained the usual amount of $Ca^{2+}$ (1.8 mM), or $Ca^{2+}$ was depleted by addition of magnesium EGTA to an excess of 2 mM. After 3 h of incubation, the amount of cell-associated radioactivity was measured. The association is expressed as percentage of the association at 1.8 mM $Ca^{2+}$ (364 ± 21 ng of apolipoprotein mg$^{-1}$ of cell protein for [$^{125}$I]LDL and 10.7 ± 0.2 μg of phospholipid mg$^{-1}$ cell protein for radiolabelled apoE liposomes). Values are means ± s.d. of four experiments

| | Association (% of control) | |
|---|---|---|
| | ApoE liposomes | LDL |
| 1.8 mM $Ca^{2+}$ | 100 ± 2 | 100 ± 6 |
| 2 mM Magnesium EGTA excess | 15 ± 2 | 29 ± 2 |

**Figure 4** Serum clearance of apoE-containing LAD-liposomes in B16 tumour-bearing mice. B16 tumour-bearing mice were intravenously injected with LAD-loaded apoE-liposomes, labelled with [$^{3}$H]CO-ether (■) and [$^{14}$C]CO-ester (▨), at a dose of 0.5 mg of phospholipid per mouse. Mice were sacrificed at 2, 4 or 8 hours after injection, and the radioactivity in the serum was measured. Results are expressed as percentage of the injected dose recovered in the serum. Values are means ± SD of 3–5 animals

**Figure 5** Uptake of apoE-enriched LAD-liposomes by liver, adrenals, spleen and tumour in B16 tumour-bearing mice. B16 tumour-bearing mice were intravenously injected with LAD-loaded apoE-liposomes, labelled with [$^{3}$H]CO-ether (■) and [$^{14}$C]CO-ester (▨), at a dose of 0.5 mg of phospholipid per mouse. Mice were sacrificed at 2, 4 or 8 hours after injection, and the radioactivity in the liver, adrenals, spleen and tumour was measured. The results are expressed as the percentage of the injected dose that was recovered per gram of tissue and are means ± SD of 3–5 animals the association and the degradation of [$^{125}$I]LDL by B16 cells. Association of [$^{125}$I]LDL was reduced by 50% in the presence of 50 nM of LDL. However, only 1.7 nM apoE-enriched liposomes was sufficient to obtain a similar competition. Compared with the apoE liposomes, liposomes lacking apoE were approximately 20-fold less effective in competing with [$^{125}$I]LDL for association by B16 cells. The degradation of [$^{125}$I]LDL was inhibited even more efficiently by apoE-enriched liposomes: 50% inhibition was observed after addition of 0.8 nM of apoE liposomes. LDL and liposomes without apoE were less effective.

Table 1 summarizes the results of a competition study in which B16 cells were incubated with 1.6 nM of radioactively labelled apoE-enriched liposomes (approximately twice the $K_d$) in the presence of increasing amounts of unlabelled apoE-enriched liposomes, liposomes or LDL. The results resemble the above-mentioned findings with iodinated LDL as radiolabelled substrate. LDL and unlabelled apoE-enriched liposomes were both able to inhibit the association of the radiolabelled apoE-enriched liposomes. However, it took 600 nM of LDL to displace 60% of the associated apoE-enriched liposomes, whereas only 30 nM of unlabelled apoE-enriched liposomes sufficed to inhibit the association of the labelled apoE-enriched liposomes by 90%. The addition of an equal amount of liposomes without apoE had no significant effect on the association of the labelled apoE-enriched liposomes. We conclude from the cross-competition studies that the interaction of apoE-enriched liposomes with B16 cells is LDL-receptor mediated. Our results further indicate that the presence of apoE is crucial for the interaction of the liposomes with B16 cells.

### Interaction of apoE-enriched liposomes with cultured B16 cells: $Ca^{2+}$-depletion studies

The binding of LDL to the 'classical' LDL receptor is strictly $Ca^{2+}$ dependent (Goldstein and Brown, 1974). We showed recently that the interaction of LDL with B16 cells is also strongly $Ca^{2+}$ dependent (Versluis et al, 1996). Table 2 demonstrates that the association of apoE-enriched liposomes with B16 cells is, like LDL, strongly diminished by depletion of $Ca^{2+}$, which indicates that the LDL receptor is involved in the interaction of apoE-enriched liposomes with B16 cells.

© Cancer Research Campaign 1998

**Figure 6** Tissue distribution of LAD-loaded apoE-liposomes. B16 tumour-bearing mice were intravenously injected with LAD-loaded apoE-liposomes, labelled with either [³H]CO-ether (■) or [³H]LAD (▨), at a dose of 0.5 mg of phospholipid per mouse. Eight hours after injection, a 0.4 ml blood sample was taken and the mice were sacrificed. Radioactivity was measured in the indicated organs and tissues. Results are expressed as the percentage of the injected dose that was recovered per gram of tissue, and are means ± SD of 2 or 5 animals ([³H]LAD or [³H]CO-ether label, respectively)

**Figure 7** Tissue distribution of [³H]CO-ether labelled LAD-loaded apoE-liposomes and [¹²⁵I]TC-LDL in B16 tumour-bearing mice. B16 tumour-bearing mice were intravenously injected with [³H]CO-ether labelled LAD-loaded apoE-liposomes (■: 0.5 mg of phospholipid per mouse) or with [¹²⁵I]tyramine cellobiose-labelled LDL (▨: 40 μg of apolipoprotein per mouse). Mice were sacrificed 24 h (LDL injection) or 8 h (liposomes injection) later, and radioactivity was measured in the indicated organs and tissues. Results are expressed as the percentage of the injected dose that was recovered per gram of tissue, and are means ± SD of 3 or 5 animals ([¹²⁵I]TC-LDL or [³H]CO-ether liposomes, respectively)

### Disposition of apoE-enriched liposomes and liposome-associated LAD in mice bearing B16 tumours

To investigate in vivo the tumour-localizing properties of apoE-enriched liposomes and their ability to deliver drugs to tumours, we incorporated LAD, a lipophilic prodrug of the anti-tumour drug daunorubicin, into the liposomes. Subsequently, we examined the fate of the apoE liposomes and the prodrug in mice bearing a B16 tumour.

Initially, we studied the processing of the apoE-enriched LAD liposomes. The liposomes were labelled with both a biodegradable label ([¹⁴C]cholesteryl oleate ester) and a non-degradable label ([³H]cholesteryl oleate ether). The radiolabelled liposomes were injected into the tumour-bearing mice, and the serum clearance of both labels and their accumulation in several LDL receptor-expressing organs (i.e. liver, adrenals and spleen) and in tumour tissue were monitored. The serum clearance, presented in Figure 4, was similar for both labels. The amount of non-degradable label in liver, adrenals and spleen, which represents the absolute uptake of the apoE-enriched LAD liposomes, increased with the circulation time (Figure 5). The amount of degradable label in these tissues, however, was much lower. This indicates that the LAD-loaded apoE liposomes are internalized and processed in tissues, followed by excretion of the radiolabelled degradation products. ApoE-enriched LAD liposomes also accumulated in the tumour. However, no significant amounts of the biodegradable label were excreted from the tumour, not even after 8 h.

To investigate in vivo the tumour uptake of a liposome-associated drug, [³H]LAD was incorporated into liposomes. The drug–carrier complex was provided with apoE and injected into B16 tumour-bearing mice. Figure 6 shows the distribution of [³H]LAD radioactivity over tissues, which was determined 8 h later. To monitor the fate of the liposomal carrier, mice in a separate experiment were injected with apoE-enriched LAD liposomes that had been radioactively labelled with the non-degradable lipid [³H]cholesteryl oleate ether (represents total uptake of liposomes; Figure 6). Both distinctly labelled preparations behaved similarly in vivo, as judged from the disposition of simultaneously incorporated [¹⁴C]cholesteryl oleate ester (not shown). Eight hours after injection, the recovered amounts of LAD and apoE liposomes in serum were very similar (27% ± 2% and 30% ± 3% of the injected dose respectively). As estimated from the accumulation of the radiolabelled cholesteryl oleate ether, the liver, adrenals and the spleen showed the highest uptake of apoE-enriched liposomes (Figure 6). The tumour showed the highest uptake of all other organs. The distribution of radioactivity after injection of [³H]LAD-labelled liposomes followed the same pattern. However, the amounts of recovered radioactivity were lower, which points to metabolism of the radiolabelled prodrug.

Figure 7 compares the tissue uptake of the apoE-enriched LAD liposomal carrier with that of native LDL. Both carriers were labelled with a non-degradable label. The liposomes were labelled with [³H]CO ether and LDL with [¹²⁵I]tyramine cellobiose (TC; Pittman et al, 1983). The distributions of the labelled liposomes and LDL were determined after 8 h (70% ± 3% of the dose cleared from the serum) and 24 h (85% ± 3% of the dose cleared) respectively. It should be noted that after uptake of [¹²⁵I]TC-labelled LDL by the liver, part of the [¹²⁵I]TC radiolabel is excreted via the bile into the intestines (Kleinherenbrink-Stins et al, 1990). Therefore, the amount of TC label in the intestines should be added to the amount in the liver. Taking this [¹²⁵I]TC transfer into consideration, the uptake pattern of the LAD-loaded apoE-enriched

© Cancer Research Campaign 1998

liposomes by the organs and the tissues shows a striking similarity with that of LDL, which strongly suggests the involvement of the LDL receptor in the uptake of LAD-loaded apoE-enriched liposomes.

### DISCUSSION

We recently synthesized small apoE-containing liposomes, with the aim of using these particles for the selective delivery of antineoplastic agents to tumours via the LDL receptor. The apoE liposomes behaved in vivo similarly to native LDL (i.e. no significant uptake by the reticuloendothelial system and metabolic fate dependent on the level of LDL-receptor expression; Rensen et al, 1997). We further incorporated LAD, a lipophilic prodrug of daunorubicin, into these liposomes (approximately ten prodrug molecules per particle; Versluis et al, 1998). In the present study, we investigated the interaction of apoE-containing liposomes with cultured B16 melanoma cells, and we studied the tumour-localizing properties of LAD-loaded apoE-liposomes in the B16 tumour–mouse model.

The binding of apoE liposomes to cultured B16 cells was saturable, indicating receptor-mediated binding. Cross-competition studies with LDL indicate that apoE liposomes are specifically bound by LDL receptors on B16 cells. Further evidence for implication of LDL receptors comes from the finding that association of apoE liposomes with B16 cells is $Ca^{2+}$ dependent, a classical characteristic of the LDL receptor (Goldstein and Brown, 1974). The essential role of apoE for the recognition of the liposomes by the LDL receptor became evident from the finding that liposomes lacking apoE are far less effective in competing for receptor binding. ApoE liposomes bind to the LDL receptor with a 15-fold higher affinity ($K_d$ 0.77 nM) than LDL ($K_d$ 11.5 nM). The number of binding sites for apoE liposomes is, however, two- to 2.5-fold lower than that for LDL (90 000 versus 220 000). These results are in agreement with the findings of Pitas et al (1980), who showed that lipid vesicles provided with increasing numbers of apoE molecules displayed an increasing affinity for the LDL receptor. The maximum affinity, which was 25-fold higher than that of LDL, was reached when at least four apoE molecules were present per particle. At this point, the maximal binding capacity was decreased fourfold, indicating multivalent binding of the apoE-containing particles to LDL receptors.

The tumour-localizing properties of the apoE liposomes were studied by administering LAD-loaded apoE-liposomes to B16 tumour-bearing mice. To study processing, the liposomes were double-labelled with a non-degradable lipid label (represents total amount taken up) and a biodegradable label. The latter can be metabolized and excreted, and gives an indication of the processing of the particles. We found that the particles are not processed in the circulation, but were taken up and rapidly processed by the three organs with a high expression of the LDL receptor (i.e. liver, adrenals and spleen). Thus, the prodrug-loaded liposomes are, like native LDL, internalized and processed. Of all other organs, the tumour showed the highest uptake. However, we detected no significant difference in accumulation of the labels in the tumour. This finding does not necessarily mean that the liposomes are not processed by the tumour. The liver and adrenals use cholesterol for the synthesis of secretion products (bile salts, corticosteroid hormones). Tumours, however, use the cholesterol to synthesize cellular components that remain cell-associated. Further, earlier results indicate that LDL is processed by the B16 tumour inoculated in mice, albeit at a somewhat lower rate than in liver and adrenals (Versluis et al, 1996). The tissue distribution of LAD-loaded apoE-liposomes is very similar to that of native LDL, which further supports the involvement of the LDL receptor in the uptake of LAD-loaded apoE liposomes.

The ability of the apoE liposomes to deliver LAD to the tumour was studied by injecting [$^3$H]LAD-loaded apoE liposomes. The serum clearance of the radiolabelled prodrug was similar to the serum clearance of the radioactivity after injection of LAD-loaded [$^3$H]CO-labelled apoE liposomes, indicating a concomitant clearance and uptake of drug and carrier. The recovery of LAD in the tissues was much lower than that of the non-degradable liposomal label, which points to processing of the prodrug. This suggests that, as anticipated, daunorubicin is released from the tetrapeptide spacer and is further metabolized. Assuming that all incorporated LAD is delivered to the tumour, and that tumour cells constitute 10% of the tumour weight (Murray and Carmichael, 1995), it can be calculated that approximately 0.2 μmol of LAD can be delivered per litre of B16 cell volume at the present dosing. The effective intracellular anthracycline concentration, which is in the μM range (Speth et al, 1988), should easily be reached by higher and more frequent dosing.

Uptake of the prodrug-loaded liposomes by liver, spleen and adrenals is higher than the uptake by the tumour. If a therapeutic dose is to be targeted to the tumour, precautions are necessary to protect these organs from irreversible tissue damage. Expression of LDL receptors in the liver can be decreased by a diet enriched in cholesterol and triglycerides rich in saturated fatty acids (Angelin et al, 1983; Dietschy et al, 1993; Packard et al, 1983). The expression of LDL receptors in the spleen and adrenals can be significantly reduced by the administration of bile salts and corticosteroids respectively (Hynds et al, 1984; Isaacsohn et al, 1986).

In conclusion, apoE liposomes, prepared from commercially available lipids and recombinant apoE, can be used as carriers for LDL receptor-mediated delivery of drugs to tumours. The particles have a much higher affinity for the LDL receptor than native LDL, which affords a competitive advantage. The liposomes have the capacity to incorporate lipophilic anti-tumour (pro)drugs in the phospholipid bilayer and water-soluble drugs in the aqueous core, which provides numerous possibilities to further develop the drug-disposing properties of the apoE-enriched liposomes. ApoE-enriched liposomes are, thus, a new option and stimulus for the application of a LDL receptor-mediated tumour therapy.

### REFERENCES

Angelin B, Raviola CA, Innerarity TL and Mahley RW (1983) Regulation of hepatic lipoprotein receptors in the dog. *J Clin Invest* **71**: 816–831

Bilheimer DW, Eisenberg S and Levy RI (1972) The metabolism of very low density lipoproteins. I. Preliminary in vitro and in vivo observations. *Biochim Biophys Acta* **280**: 212–221

Brown MS and Goldstein JL (1986) A receptor-mediated pathway for cholesterol homeostasis. *Science* **232**: 34–47

De Smidt PC and Van Berkel ThJC (1990) Prolonged serum half-life of antineoplastic drugs by incorporation into the low density lipoprotein. *Cancer Res* **50**: 7476–7482

Dietschy JM, Turley SD and Spady DK (1993) Role of liver in the maintenance of cholesterol and low density lipoprotein homeostasis in different animal species, including humans. *J Lipid Res* **34**: 1637–1659

Firestone RA (1994) Low-density lipoprotein as a vehicle for targeting antitumor compounds to cancer cells. *Bioconjugate Chem* **5**: 105–113

Firestone RA, Pisano JM, Falck JR, McPhaul MM and Krieger M (1984) Selective delivery of cytotoxic compounds to cells by the LDL pathway. *J Med Chem* **27**: 1037–1043

© Cancer Research Campaign 1998

Goldstein JL and Brown MS (1974) Binding and degradation of low density lipoproteins by cultured human fibroblasts. *J Biol Chem* **249**: 5153–5162

Ho YK, Smith RG, Brown MS and Goldstein JL (1978) Low-density lipoprotein (LDL) receptor activity in human acute myelogenous leukemia cells. *Blood* **52**: 1099–1114

Hynds SA, Welsh J, Stewart JM, Jack A, Soukop M, McArdle CS, Calman KC, Packard CJ and Shepherd J (1984) Low-density lipoprotein metabolism in mice with soft tissue tumours. *Biochim Biophys Acta* **795**: 589–595

Innerarity TL and Mahley RW (1978) Enhanced binding by cultured human fibroblasts of apo-E-containing lipoproteins as compared with low density lipoproteins. *Biochemistry* **17**: 1440–1447

Innerarity TL, Pitas RE and Mahley RW (1979) Binding of arginine-rich (E) apoprotein after recombination with phospholipid vesicles to the low density lipoprotein receptors of fibroblasts. *J Biol Chem* **254**: 4186–4190

Isaacsohn JL, Lees AM, Lees RS, Strauss HW, Barlai-Kovach M and Moore TJ (1986) Adrenal imaging with technetium-99m-labelled low density lipoproteins. *Metabolism* **35**: 364–366

Kleinherenbrink-Stins MF, Van Der Boom J, Bakkeren HF, Roholl PJM, Brouwer A, Van Berkel ThJC and Knook DL (1990) Light- and immunoelectron microscopic visualization of in vivo endocytosis of low density lipoprotein by hepatocytes and Kupffer cells in rat liver. *Lab Invest* **63**: 73–86

Lowry OH, Rosebrough NJ, Farr AL and Randall RJ (1951) Protein measurement with the Folin phenol reagent. *J Biol Chem* **193**: 265–275

Lundberg B and Suominen L (1984) Preparation of biologically active analogs of serum low density lipoprotein. *J Lipid Res* **25**: 550–558

Lundberg B, Hong K and Papahadjopoulos D (1993) Conjugation of apolipoprotein B with liposomes and targeting to cells in culture. *Biochim Biophys Acta* **1149**: 305–312

Masquelier M, Vitols S and Peterson C (1986) Low-density lipoprotein as a carrier of antitumoral drugs: in vivo fate of drug–human low-density lipoprotein complexes in mice. *Cancer Res* **46**: 3842–3847

Mosley ST, Goldstein JL, Brown MS, Falck JR and Anderson RGW (1981) Targeted killing of cultured cells by receptor-dependent photosensitization. *Proc Natl Acad Sci USA* **78**: 5717–5721

Murray JC and Carmichael J (1995) Targeting solid tumours: challenges, disappointments and opportunities. *Adv Drug Deliv Rev* **17**: 117–127

Packard CJ, McKinney L, Carr K and Shepherd J (1983) Cholesterol feeding increases low density lipoprotein synthesis. *J Clin Invest* **72**: 45–51

Pitas RE, Innerarity TL, Arnold KS and Mahley RW (1979) Rate and equilibrium constants for binding of apo-E $HDL_c$ (a cholesterol-induced lipoprotein) and low density lipoproteins to human fibroblasts: evidence for multiple receptor binding of apo-E $HDL_c$. *Proc Natl Acad Sci USA* **76**: 2311–2315

Pitas RE, Innerarity TL and Mahley RW (1980) Cell surface receptor binding of phospholipid protein complexes containing different ratios of receptor-active and -inactive E apolipoprotein. *J Biol Chem* **255**: 5454–5460

Pittman RC, Carew TE, Glass CK, Green SR, Taylor CA and Attie AD (1983) A radioiodinated, intracellularly trapped ligand for determining the sites of plasma protein degradation in vivo. *Biochem J* **212**: 791–800

Ponty E, Favre G, Benaniba R, Boneu A, Lucot H, Carton M and Soula G (1993) Bio-distribution study of $^{99m}$Tc-labelled LDL in B16-melanoma-bearing mice. Visualization of a preferential uptake by the tumor. *Int J Cancer* **54**: 411–417

Redgrave TG, Roberts DCK and West CE (1975) Separation of plasma lipoproteins by density-gradient ultracentrifugation. *Anal Biochem* **65**: 42–49

Rensen PCN, Schiffelers RM, Versluis AJ, Bijsterbosch MK, Meuwissen MEMJ and Van Berkel ThJC (1997) Human recombinant apolipoprotein E-enriched liposomes can mimic low density lipoproteins as carriers for the site-specific delivery of anti-tumour agents. *Mol Pharmacol* **52**: 445–455

Samadi-Baboli M, Favre G, Bernadou J, Berg D and Soula G (1990) Comparative study of the incorporation of ellipticine-esters into low density lipoprotein (LDL) and selective cell uptake of drug-LDL complex via the LDL receptor pathway in vitro. *Biochem Pharmacol* **40**: 203–212

Samadi-Baboli M, Favre G, Canal P and Soula G (1993) Low density lipoprotein for cytotoxic drug targeting: improved activity of elliptinium derivative against B16 melanoma in mice. *Br J Cancer* **68**: 319–326

Speth PAJ, Van Hoesel QGCM and Haanen C (1988) Clinical pharmacokinetics of doxorubicin. *Clin Pharmacokinet* **15**: 15–31

Tokui T, Tokui Y, Ishigami M, Tanzawa K, Ikeda T and Komai T (1994) Targeting of an antitumor agent, RS-1541 (palmitoyl-rhizoxin), via low-density lipoprotein receptor. *Int J Pharm* **110**: 277–283

Van Berkel ThJC, Kruijt JK, Van Gent T and Van Tol A (1981) Saturable high affinity binding, uptake and degradation of rat plasma lipoproteins by isolated parenchymal and non-parenchymal cells from rat liver. *Biochim Biophys Acta* **665**: 22–33

Versluis AJ, Van Geel PJ, Oppelaar H, Van Berkel ThJC and Bijsterbosch MK (1996) Receptor-mediated uptake of low-density lipoprotein by B16 melanoma cells in vitro and in vivo in mice. *Br J Cancer* **74**: 525–532

Versluis AJ, Rump ET, Rensen PCN, Van Berkel ThJC and Bijsterbosch MK (1998) Synthesis of a lipophilic daunorubicin derivative and its incorporation into lipidic carriers developed for LDL receptor-mediated tumor therapy. *Pharm Res* **15**: 531–537

Vitols SG, Masquelier M and Peterson CO (1985) Selective uptake of a toxic lipophilic anthracycline derivative by the LDL receptor pathway in cultured fibroblasts. *J Med Chem* **28**: 451–454

Vogel T, Weisgraber KH, Zeevi MI, Ben-Artzi HB, Levanon AZ, Rall Jr. SC, Innerarity TL, Hui DY, Taylor JM, Kanner D, Yavin Z, Amit B, Aviv H, Gorecki M and Mahley RW (1985). Human apolipoprotein E expression in *Escherichia coli*: structural and functional identity of the bacterially produced protein with plasma apolipoprotein E. *Proc Natl Acad Sci USA* **82**: 8696–8700

© Cancer Research Campaign 1998